# La Niña weather impacts dietary patterns and dietary diversity among children in the Peruvian Amazon

**DOI:** 10.1017/S1368980020003705

**Published:** 2021-08

**Authors:** Ramya Ambikapathi, Margaret N Kosek, Gwenyth O Lee, Maribel Paredes Olortegui, Benjamin Zaitchik, Pablo Peñataro Yori, Aubrey Bauck, Laura E Caulfield

**Affiliations:** 1 Department of International Health, The Johns Hopkins Bloomberg School of Public Health, Baltimore, MD 21205, USA; 2 Biomedical Investigations Unit AB PRISMA, Iquitos, Peru; 3 Division of Infectious Diseases and International Health & Public Health Sciences, University of Virginia, Charlottesville, USA; 4 Department of Epidemiology, University of Michigan, Ann Arbor, USA; 5 Department of Earth and Planetary Science, The Johns Hopkins University, Baltimore, USA

**Keywords:** Climate change, Peru, ENSO, Diets, Amazon, Nutrition, Children

## Abstract

**Objective:**

In 2011–2012, severe El Niño Southern Oscillation (ENSO) conditions (La Niña) led to massive flooding and temporarily displacement in the Peruvian Amazon. Our aims were to examine the impact of this ENSO exposure on child diets, in particular: (1) frequency of food consumption patterns, (2) the amount of food consumed (g/d), (3) dietary diversity (DD), (4) consumption of donated foods, among children aged 9–36 months living in the outskirts of City of Iquitos in the Amazonian Peru.

**Design:**

This was a longitudinal study that used quantitative 24-h recall dietary data collection from children aged 9–36 months from 2010 to 2014 as part of the MAL-ED birth cohort study.

**Setting:**

Iquitos, Loreto, Peru.

**Participants:**

Two hundred and fifty-two mother–child dyads.

**Results:**

The frequency of grains, rice, dairy and sugar in meals reduced by 5–7 %, while the frequency of plantain in meals increased by 24 % after adjusting for covariates. ENSO exposure reduced girl’s intake of plantains and sugar. Despite seasonal fluctuations in the availability of fruits, vegetables and fish, DD remained constant across seasons and as children aged. However, DD was significantly reduced under moderate La Niña conditions by 0·32 (*P* < 0·05) food groups. Adaptive social strategies such as consumption of donated foods were significantly higher among households with girls.

**Conclusions:**

This is the first empirical study to show differential effect of the ENSO on the dietary patterns of children, highlighting differences by gender. Public health nutrition programmes should be climate- and gender-sensitive in their efforts to safeguard the diets of vulnerable populations.

El Niño Southern Oscillation (ENSO) is a naturally occuring, inter-annual weather pattern that results from oceanic–atmospheric interaction in the Pacific Ocean, and it affects temperature and precipitation patterns around the world^(1–4)^. During the El Niño phase, global mean temperatures are higher and vice versa in the La Niña phase^(4)^. Although the phases of ENSO (El Niño and La Niña) occur over 2–7 years cycles, there is growing concern that the variability and severity of ENSO cycles are associated with a rise in global temperature due to climate change^(1,5)^. ENSO events are associated with droughts in South East Asia and in Southern Africa, floods in the Amazon, and hurricanes in the Carribean and in the Gulf of Mexico^(6–8)^. The resulting variabilities in crop yields are in different directions depending upon the ENSO (El Niño *v*. La Niña) phase and have differential effects around the world. For example, the El Niño phase can cause torrential rainfall and flooding in coastal Peru, leading to crop failure through excess soil saturation and mudslides, whereas in the interior Amazon, South East Asia, Malaysia, and Indonesia, crop failures occur due to reduced rainfall and drought-like conditions^(9)^.

In Peru, the effects of ENSO on food systems have been documented during each cycle since the 1950s. During the 1972 El Niño, catastrophic damage to the coastal fisheries led to a nationalisation of the fishing industry. The record-breaking El Niño cycle in 1982–83 caused some regions of Peru to receive ‘seven years of worth of rain in four months’^(10)^, resulting in heavy damage to infrastructure and crop yields. Crop failures were widespread, with rice, potatoes, cotton, sugar cane and alfalfa losses valued at USD 244 million (USD 596 million in 2013)^(10)^. This was termed an ‘environmental-ecological crisis’ due to cyclical damages to infrastructure, health and the economy^(10)^. In the 1997–98 ENSO cycle, the coastal region of Peru reported 3300 millimetres (mm) of rain when the average rainfall is usually less than 200 mm^(11)^.

In the Peruvian Amazon, annual river discharge shows a coupling pattern with the ENSO cycles – there is a lower river level during the El Niño phase coupled with low rainfall and a higher discharge during the wetter La Niña phase^(3,12)^. River discharge levels provide the main mediating link to the economy in the Amazon because flooding behaviour not only determines the biogeography of aquatic, terrestrial and human settlements but also affects food trade and transportation^(12,13)^. For example, dry/low river level season reforestation (June–November) is a period of increased availability of food for humans from both forest products and fisheries^(12,14)^. However, the wet/high river level season (December–May) is the primary driver of transfer of nutrients between terrestrial and aquatic ecosystems^(12)^. Disturbances in seasonal river flow and floods caused by ENSO have cascading effects on the interconnected ecosystem, especially on fish availability, agricultural productivity and forest products, affecting livelihoods, food availability and health outcomes^(13,15)^. In the majority of the predicted models, anthropogenic climate change is also expected to substantially increase the frequency of flooding in the Peruvian Amazon^(13)^. Understanding how these ENSO events affect dietary intakes is important for planning future mitigation strategies.

The city of Iquitos, located in northeastern Peruvian province of Loreto, is one example of a riverine community in the midst of global environmental change. The Itaya, Napa, Nanay and Amazon rivers surround the city, and the city can only be reached by air or water^(16)^. Subsistence fishing provides about 75 % of fish production in the Loreto province, in which Iquitos city is located^(17)^. Dietary intake primarily consists of seasonally available produce and animal source protein predominantly derived from fish during the dry season, but foods such as canned tuna, milk (fresh and powdered), rice and refined flours are available. River levels are the main source of transportation of food, of fish production, and access to forest products, and hence represent a critical aspect of the economy in Iquitos^(16)^.

This analysis evaluates how ENSO events translate into differences in individual-level dietary patterns, defined as the amount and diversity of foods consumed by children in Iquitos, Peru from 2010 to 2014. During the time period of data collection, El Niño and La Niña cycles of varying severity were observed, most notably severe floods were observed during the 2011–2012 La Niña^(18)^. Approximately half of the study population in these communities were temporarily displaced during this period, and the effect on dietary patterns and intake are not known. Shown in Figure 1 is the conceptual framework linking ENSO conditions to dietary intake in the Peruvian Amazon, where the effects are mediated by river level and local food prices (measured), food trade (unmeasured), and fish and food availability (unmeasured). We define the local food prices and river levels as proximal measures of climate variability and ENSO conditions as distal measure of climate variability^(14,19)^.

**Fig. 1:**
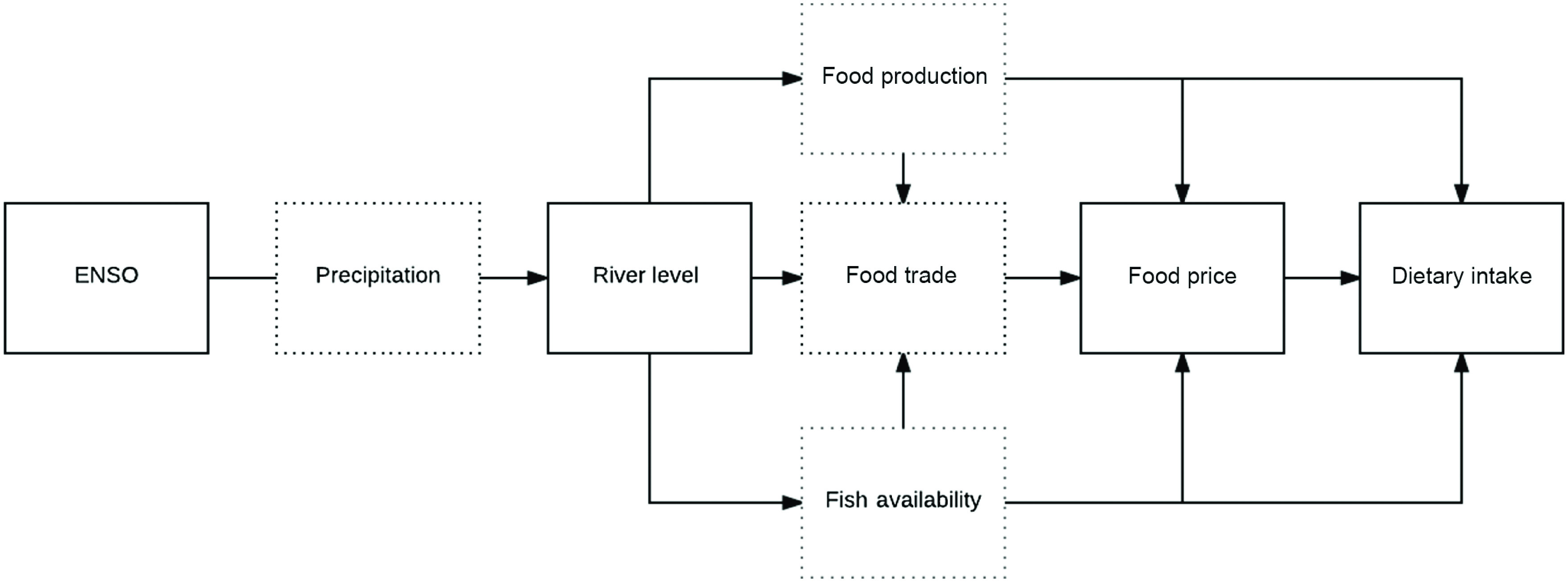
Conceptual diagram of impact of ENSO on dietary intake in the Peruvian Amazon. ENSO, El Niño Southern Oscillation

Specifically, we test (1) whether ENSO conditions (primarily La Niña in 2011–2012) led to changes in frequency of meals with fish, meat, eggs, poultry, grains, rice, plantains, yucca, dairy and sugar after adjusting for age, season, morbidity and other possible confounding factors among children aged 9–36 months in the Peruvian Amazon, and (2) whether the relationship from the first analysis was true for intake of (grams) of rice, sugar, yucca and fish consumed. Finally, we evaluate the relationship between ENSO and the dietary diversity (DD) of these children aged 9–36 months and household food insecurity coping strategies (consumption of foods donated to a household member sometimes in exchange for services) under various ENSO phases.

## Methods

### Study design

This analysis is nested within The Etiology, Risk Factors and Interactions of Enteric Infections and Malnutrition and the Consequences for Child Health and Development (MAL-ED) cohort in Peru, conducted by the co-authors^(16)^. Briefly, the MAL-ED study followed a cohort of children from birth to 36 months of age, collecting weekly and monthly data on morbidity, growth and dietary intake from eight countries^(20)^. In total, 303 mother–child pairs were enrolled from December 2009 to February 2012 from the peri-urban community of Santa Clara, situated 15 kilometers from the city of Iquitos, located in the Peruvian Amazon^(16)^. Access to government health services is reasonably high in the study population, as evidenced by high vaccination coverage in the first 12 months (> 85 %) and childbirth at health centres (> 80 %)^(16,21)^. Despite greater access to health services, this community has stunting prevalence of 45 % among children under 5 years of age, compared to the national average of 19·5 % and the regional average of 31 %^(16)^.

### Key outcome variables

Monthly dietary data and recipes were collected by trained personnel using the 24-h recall methodology from caregivers of children aged 9–36 months from August 2010 to September 2014. The rationale and methodology have previously been described^(22)^. Overall, 5716 24-h recalls and 19 035 recipes were recorded. Several outcome variables were constructed from these data to address the three aims of this report. We evaluated dietary patterns using several metrics. First, we used counts of meals or snacks with fish, meat, eggs, poultry, grains (wheat, noodle, maize and rice), rice, plantains, yucca or dairy consumed by the child. Second, we quantified the amounts in grams of rice, yucca, sugar and fish consumed by the child, and modelled intakes above zero. These were selected based on a priori hypothesis and previous research in the setting^(23)^. Fish intake is expected to be affected seasonally by river levels, and rice, yucca, and plantains are floodplain crops that are affected by river flow^(24)^. These three plant foods are interchangeably consumed as the main staple depending upon the food security of the household, with greater food insecurity associated with lower consumption of rice and greater consumption of plantains and yucca^(23)^. Third, we calculated a DD score as the sum of seven food groups (grains/root/tubers, dairy, legumes/nuts, meat, eggs, vitamin A rich fruits and vegetables, and other fruits and vegetables)^(25)^. Finally, because of prior work in the community, we included a code in the dietary recall to identify foods consumed that were donated to the individual child, which enabled us to evaluate associations between climate events and this coping strategy.

### Independent variables

The main independent measure of interest was ENSO, which is typically captured by several indices that measure temperature, precipitation and wind velocity at different locations. These indices includes Multivariate ENSO Index (MEI), Oceanic Nino Index and Southern Oscillation Index^(26)^. MEI values ranges from –3·2 to 2·6 based on exposure to La Niña (negative) and El Niño (positive) and were used here as the main exposure. Sensitivity analysis with Oceanic Nino Index and Southern Oscillation Index indices were also estimated to examine the consistency in the findings, that is, agreement with any two indices (direction and magnitude)^(27)^. Based on prior analyses of several types and classification of ENSO indices (Ambikapathi R. Effects of El Niño Southern Oscillation and Seasonality on Food Prices, Dietary and Nutrient Intake: A Case Study in Iquitos, Peru, Chapter 3 [dissertation]. Baltimore (MD): Johns Hopkins University; 2016, unpublished results) and their relationship with river discharge, a MEI severity variable with nine categories depicting the severity of ENSO events each month (neutral, weak El Niño, moderate El Niño, strong El Niño, very strong El Niño, weak La Niña, moderate La Niña, strong La Niña and very strong La Niña) was also selected to evaluate the impact of ENSO severity. The main monthly exposure, MEI index, was linked to the summary variables that had been derived from the monthly dietary recalls. Supplement Figure 1 shows relationship between dietary patterns and ENSO exposure. Daily river discharge (metres above sea level) from the Nanay River over the study period was obtained from the Sedaloreto water treatment plant located in Iquitos, Peru. Median monthly flow from 2010 to 2014 was used as a measure of local climate variability. Supplement Figure 2 shows relationship between dietary patterns and river level.

Multiple publicly available sources of data were utilised to create food price variables. Thrice weekly food price data for the Loreto region was obtained from Supply System and Prices (SISAP), published by the Peruvian Ministry of Agriculture and Irrigation (MINAGRI). From this, monthly mean prices of rice, yucca, plantain (belaco variety), white sugar, oil and eggs were calculated (in Peruvian Sol or S/. per kg, except for plantains which are per bunch) from August 2010 to September 2014. The monthly consumer price index for a food basket was obtained from the Central Reserve Bank of Peru to adjust for inflation (BCRP), and food prices were adjusted to the May 2016 consumer price index rate. Principal component analysis was conducted on the monthly mean food prices for six food items from 2010 to 2014. The first component was treated as the food price index in the models (see online supplementary material, Supplemental Table 1) and explained 64 % of the variation in the local food prices.

Child-level variables included gender and birth month-year and three time-varying covariates: age, breast-feeding (binary variable), morbidity and total energy intake. Household-level factors included a study specific socio-economic index called the water/sanitation, assets, maternal education and income index (WAMI) that consists of four components: mothers education (years), sanitation index, income (USD) and asset index^(28)^. To characterise child morbidity and adjust for potential differences in intake, we calculated the personal prevalence of diarrhoea and of illness (days with diarrhoea, fever, vomiting or cough), during the 30 d prior to the dietary recall^(29)^.

### Statistical analysis

For the first analysis, to evaluate the effects of ENSO conditions on counts of food items consumed by the child, Poisson and negative binomial regression for panel data were used. These models, which deal with over-dispersion of the data, are frequently used to examine the associations of food consumption patterns with ecological exposures such as the neighbourhood food environment^(30–32)^. Results are presented as incident rate ratios and can be interpreted as the per cent of the counts in which a specific food item was consumed (β coefficient –1 × 100 = %). Models were evaluated with or without the river flow variable to examine the magnitude of attenuation due to ENSO events. Panel regression models were used to model the consumed amount of each of the ten food items. Linear random effects models were used to examine the association between DD score and ENSO exposure because DD scores were normally distributed even across different age groups. Poisson models were estimated for consumption of donated food items. Final models were selected based on Akaike information criteria. If a model with interaction terms had a significant term and had a Akaike information criteria difference of < 10 compared to the simpler models, it was selected as the final model^(33)^. Robust standard errors were estimated to account for heteroskedasticity. Statistical significance was determined by *P* values of < 0·05; however, *P* values < 0·10 were also noted for trending significance. All analyses were performed in Stata version 13.1 (StataCorp. 2013).

## Results

Shown in Table 1 are the demographic characteristics of the MAL-ED cohort in Loreto, Peru. Among the 303 enrolled children, 46 migrated out of the study site, 4 were lost to follow-up and 1 child died before the monthly dietary intakes were quantified (which began at 9 months of age). The median number of dietary recall visits per child was 27 (interquartile range : 19, 28). The median household income of the families is USD 128 (interquartile range: 104, 170), and mothers on average had 8 years of schooling at study enrolment. Seventy-five per cent of the children were weaned by 22 months of age, and there were no significant gender differences in age at weaning.

**Table 1:** Child and family characteristics of MAL-ED cohort

	Median	Interquartile ranges (quartile 1, quartile 3)
*n*	252	
Female child (%)	45·2	
Birth order (%)		
First born	35·9	
2–4 children	47·6	
5+ children	16·5	
Length for age *z*-score (at 9 months)	–1·1	–1·8, –0·6
Weight for age *z*-score (at 9 months)	–0·4	–1·1, 0·2
Weight for height *z*-score (at 9 months)	0·8	0·1, 1·5
Dietary recalls per child from 9 to 36 months	27	19, 28
WAMI – socio-economic index		
Mother’s education (years)	8	6, 10
Sanitation index	0	0, 4
Income (USD)	127	103, 174
Assets index	5	4, 6

Table 2 shows the ENSO exposure from 2010 to 2014 by age groups. Out of 50 months, approximately half had neutral climatic conditions (42–66 % by various ENSO indices), with La Niña exposure present during 24–48 % of all months. The younger age groups experienced most of the La Niña events. The median number of days with illness reduced from eight to four days per month as children aged.

**Table 2:** Main exposure and outcomes: per cent of ENSO exposure and food consumption patterns by age group across 50 months from October 2010 to November 2014

Child age (months)	9–15 m		16–24 m		25–30 m		31–36 m		Total	
	*n*	%	*n*	%	*n*	%	*n*	%	*n*	%
Months of ENSO exposure	*n* 19		*n* 12		*n* 9		*n* 10		*n* 50	
ONI index % (# of months)										
Neutral	31·6	6	75·0	9	100·0	9	90·0	9	66·0	33
Weak La Niña	36·8	7	25·0	3	0·0	0	10·0	1	22·0	11
Moderate La Niña	31·6	6	0·0	0	0·0	0	0·0	0	12·0	6
SOI index % (# of months)										
Neutral	10·5	2	83·3	10	66·7	6	30·0	3	42·0	21
Weak El Niño	0·0	0	0·0	0	11·1	1	20·0	2	6·0	3
Weak La Niña	31·6	6	8·3	1	22·2	2	50·0	5	28·0	14
Moderate La Niña	5·3	1	8·3	1	0·0	0	0·0	0	4·0	2
Strong La Niña	26·3	5	0·0	0	0·0	0	0·0	0	10·0	5
Very strong La Niña	26·3	5	0·0	0	0·0	0	0·0	0	10·0	5
MEI index % (# of months)										
Neutral	31·6	6	50·0	6	77·8	7	70·0	7	52·0	26
Weak El Niño	0·0	0	25·0	3	22·2	2	30·0	3	16·0	8
Moderate El Niño	5·3	1	0·0	0	0·0	0	0·0	0	2·0	1
Weak La Niña	21·1	4	25·0	3	0·0	0	0·0	0	14·0	7
Moderate La Niña	5·3	1	0·0	0	0·0	0	0·0	0	2·0	1
Strong La Niña	36·8	7	0·0	0	0·0	0	0·0	0	14·0	7
River level in metres*										
Median	112		111		112		114		113	
Interquartile ranges (quartile 1, quartile 3)	110, 116		110, 116		111, 116		112, 116		110, 116	

	Median	Interquartile ranges (quartile 1, quartile 3)	Median	Interquartile ranges (quartile 1, quartile 3)	Median	Interquartile ranges (quartile 1, quartile 3)	Median	Interquartile ranges (quartile 1, quartile 3)	Median	Interquartile ranges (quartile 1, quartile 3)
Dietary recalls	*n* 1728		*n* 1897		*n* 1126		*n* 965		*n* 5716	
Children	251		225		203		187		252	
Meals with fish	0	0, 1	0	0, 1	0	0, 1	0	0, 1	0	0, 1
Meals with grains	4	3, 5	4	3, 6	4	3, 6	4	3, 5	4	3, 5
Meals with meat	0	0, 1	1	0, 2	1	0, 2	1	0, 2	1	0, 2
Meals with egg	1	0, 1	1	0, 2	1	0, 1	1	0, 1	1	0, 1
Meals with yucca	0	0, 0	0	0, 0	0	0, 0	0	0, 0	0	0, 0
Meals with chicken	0	0, 1	1	0, 2	1	0, 2	1	0, 2	1	0, 2
Meals with plantains	0	0, 0	0	0, 0	0	0, 1	0	0, 1	0	0, 1
Meals with rice	2	1, 2	2	1, 3	2	2, 3	2	2, 3	2	1, 3
Meals with dairy	1	0, 2	1	0, 2	1	0, 3	1	0, 2	1	0, 2
Meals with sugar	2	1, 3	2	1, 3	2	1, 4	2	1, 4	2	1, 3
Yucca consumed in grams	0	0, 0	0	0, 0	0	0, 0	0	0, 0	0	0, 0
Fish consumed in grams	0	0, 10	0	0, 23	0	0, 35	0	0, 38	0	0, 22
Rice consumed in grams	23	11, 40	38	23, 62	54	31, 87	62	39, 95	39	20, 67
Sugar consumed in grams	19	6, 36	36	17, 66	58	27, 100	62	29, 106	35	15, 72
Energy (kcals)*	440	290, 647	867	627, 1122	1133	877, 1466	1215	967, 1548	853	531, 1195
Illness last 30 d	8	3, 14	6	1, 11	5	1, 12	4	0, 10	6	1, 12

ENSO, El Niño Southern Oscillation; ONI, Oceanic Niño Index; SOI, Southern Oscillation Index; MEI, Multivariate ENSO Index.

* To convert energy values from kilocalories to kilojoules, multiply by 4·184.

### Meal consumption and local food prices

**Fig. 2:**
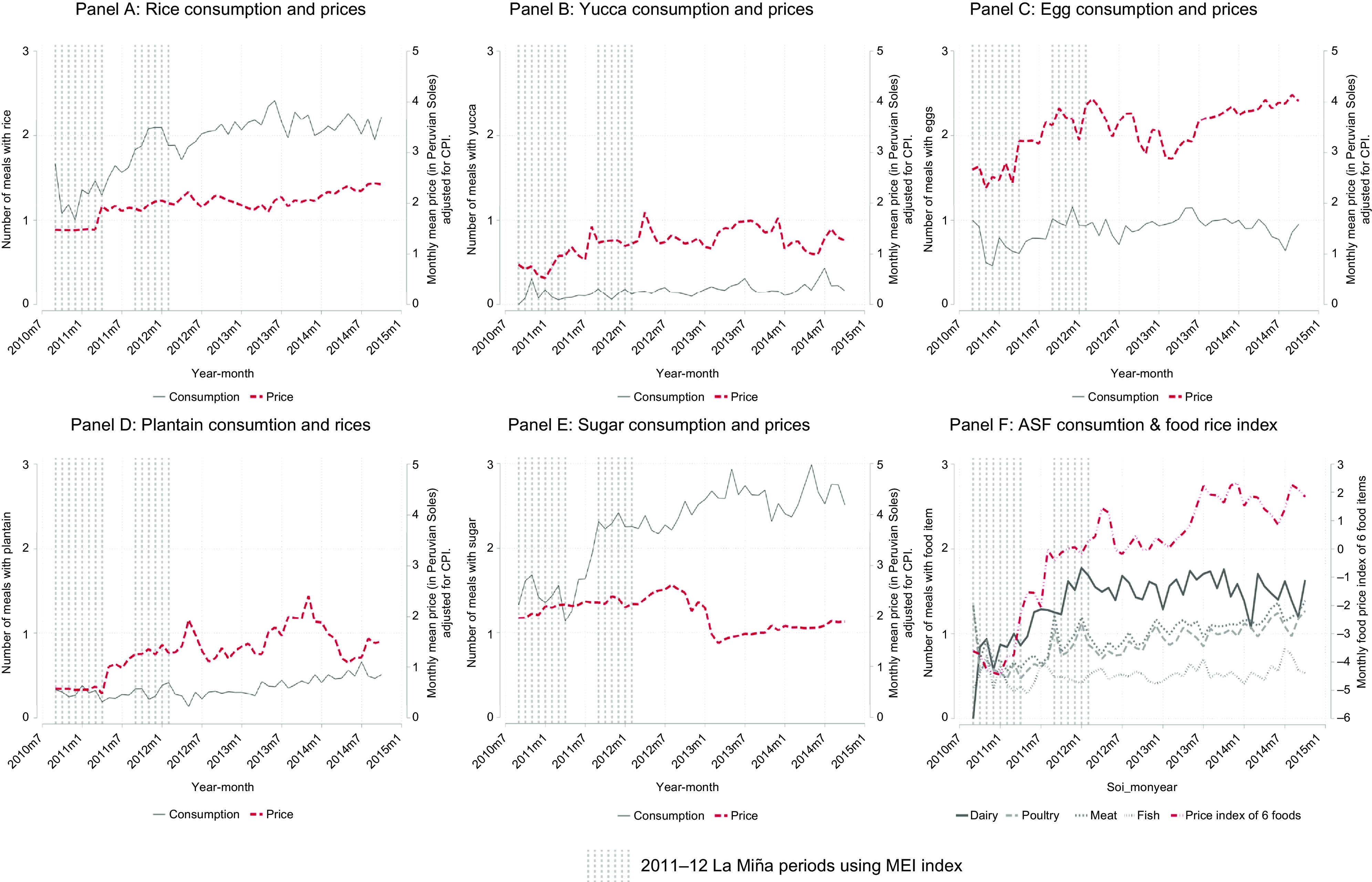
Relationship between dietary patterns of children aged 9–36 months and food prices of rice, yucca, egg, plantain and sugar (in Peruvian Soles, adjusted for consumer price index [CPI]) overlaid with periods of 2011–2012 La Niña phase from the Multivariate ENSO Index (MEI) index

Overall, children received an average of two meals with rice, four meals with grains (includes rice, wheat products and other staples), and one meal with eggs and dairy per day. It is important to note that these meals can be inclusive of other ingredients, for example, if a child consumes a breakfast meal with rice and beans with eggs, this would be counted as one meal with rice, one meal with beans and one meal with eggs. In Figure 2, food consumption and food prices are shown from July 2010 to September 2014. Eggs (Panel C) and dairy (Panel F) were the most commonly consumed animal sources of protein, with poultry and overall meat intake increasing with age.

Prior to constructing models to examine the relationship between ENSO phase and diet, we formally explored seasonal trends. Seasonality reflects within-year fluctuation that is distinct from ENSO, but seasonal trends are exacerbated by ENSO. As a result, adjusting for seasonality in our model allows us to examine the impact on ENSO on top of, or in addition to, normal seasonal trends. We observed seasonal shifts in animal source protein (Panel F). Small spikes in fish consumption were observed from June to September each year corresponding to lower river levels. Poultry intake was higher from September to January of each year. Dairy intake spiked every 3 months across all years (Fig. 1). During months with greater fish intake, fewer meals with meat were consumed. There were no significant seasonal trends in consumption of rice or plantains. Consumption of all animal source foods other than fish and eggs were strongly and positively associated with ownership of assets, whereas fish, rice, plantains and yucca consumption were associated negatively with ownership of assets. More days with illness was significantly yet minimally associated with reduced intake of meat.

Overall, the three staples, especially rice, show the least variation in prices compared to eggs, oil and sugar from 2010 to 2014. Plantain and yucca prices increased after the two La Niña periods, and some of these effects were concurrent with dips in child consumption. A price spike in July 2012 corresponded to a dip in plantain consumption (Panel D) and a decrease in egg prices in January 2012 corresponded to a rise in the consumption of meals with eggs (Panel C). Sugar prices were fairly stable until January 2013, where a drop in price and a corresponding increase in consumption were observed. This dip in prices is attributed to a consolidation of sugar companies and optimal weather conditions^(34)^.

The multivariate models shown in Table 3 confirm that grains, plantains, rice, dairy and sugar consumption were reduced under ENSO conditions. These results are consistent across at least two ENSO indices for grains, plantains and rice. There was no effect of ENSO on the frequency of meals with fish or any other animal source of protein. Model sets 2 and 3 show results with additional local-level climate variability – food prices and river-level covariates – included (see online supplementary material, Supplemental Table 2). From model set 2, we see much of the association of ENSO on food consumption was attenuated by the inclusion of local food prices in the model, suggesting that food prices are mediators of the climate–consumption relationship. This was particularly true for rice. In Supplemental Table 1, coefficients for the local food price index are shown. Notably, 1 sd in the local food prices index was associated with 2–3 % differences in intake of rice and eggs. Minor attenuation of ENSO coefficient were also observed by including river level in the model (see model set 3 in Table 3) . Model set 4 in Table 3 presents the interaction term between ENSO phase and child gender on frequency of food items. We find that girls are consuming sugar and plantains less frequently than boys by 10–24 % (*P* < 0·05).

**Table 3:** Poisson panel regression results examining ENSO exposure on food items consumed by children aged 9–36 months. All models adjusted for gender, age, parity, seasons (months), assets, energy (kcal), household income, maternal education and illness in the previous 30 d. In addition, model set 2 adjusted for local food prices (index) of six foods. Model set 3 adjusted additional for median river level (metres). Model set 4 presents the interaction term between ENSO pphases and gender and also adjusted for river levels (metres)

Model set 1	Fish	Grains	Meat	Eggs	Yucca	Chicken	Plantains	Rice	Dairy	Sugar
ONI (La Nina)	1·036	0·958*	1·029	0·986	0·882	1·043	1·246**	0·938*	0·932*	0·941*
SOI (La Nina)	1·053	1·011	1·032	1·043	0·972	1·017	1·201***	0·988	0·979	0·986
MEI (La Nina)	0·969	0·963*	1·055	0·972	1·045	1·056	1·195*	0·942*	0·981	0·971
Model set 2 with local food price index										
ONI (La Nina)	1·038	0·961*	1·031	0·992	0·894	1·045	1·242*	0·946†	0·931*	0·942*
SOI (La Nina)	1·055	1·015	1·034	1·05	0·981	1·019	1·198*	0·995	0·98	0·989
MEI (La Nina)	0·97	0·969	1·06	0·982	1·076	1·062	1·187*	0·954	0·982	0·975
Model set 3 with river level										
ONI (La Nina)	1·035	0·957*	1·029	0·983	0·886	1·043	1·227**	0·938*	0·934†	0·942*
SOI (La Nina)	1·052	1·012	1·032	1·042	0·976	1·017	1·195***	0·989	0·979	0·987
MEI (La Nina)	0·969	0·962*	1·056	0·973	1·042	1·058	1·213**	0·940*	0·975	0·968
Model set 4 interaction with gender, adjusted for river level										
ONI × Female	1·049	1·002	0·983	1·046	0·941	0·978	0·766*	1·031	1·017	0·903*
SOI × Female	1·01	1·002	0·994	0·956	0·801	0·988	0·815*	1·005	1·077	0·911*
MEI × Female	1·048	0·984	0·912	1·052	1·447†	0·878†	0·837	0·998	1·039	0·903*

ENSO, El Niño Southern Oscillation; ONI, Oceanic Niiño Index; SOI, Southern Oscillation Index; MEI, Multivariate ENSO Index.

Exponentiated coefficients; †*P* < 0·10; *P < 0·05; **P < 0·01; ***P < 0·001.

### Amount consumed

Shown in Table 4 are the regresison results of amounts consumed in grams of the four food items. Grams of rice, fish and sugar consumed significantly increased as children aged. Rice and sugar were the mostly commonly consumed items, with up to 94·1 % and 87·1 % of the dietary recalls having one meal/snack with rice or sugar. The food least commonly consumed among the four (rice, yucca, sugar and fish) food items was yucca, present in only 13·0 % of the recalls (child days). Fish consumption was reported in 38·0 % of the recalls and reflected the seasonal availability of fish, except for canned tuna. The top five most commonly consumed fish species were palometa (*Mylosoma duriventris*), boquichico (*Prochilodus nigricans*), bagre or flat-whiskered catfish (*Pinirampus pirinampu*), canned tuna and tilapia (*Tilapia rendalli*).

**Table 4:** Panel regression of ENSO exposure in intake of fish, yucca, plants and rice in grams. All models adjusted for gender, age, parity, seasons (months), assets, energy (kcal, to convert energy values from kilocalories to kilojoules, multiply by 4·184), household income, maternal education and illness in the previous 30 d. Model set 2 adjusted additionally for median river level (metres). Model set 3 presents the interaction term and also adjusted for river levels (metres)

Model set 1: Adjusted for all covariates	Yucca	Fish	Rice	Sugar
ONI (La Nina)	–7·384*	0·517	–2·632†	0·0177
SOI (La Nina)	–7·165*	–4·719*	0·978	–2·372
MEI (La Nina)	–3·287	–3·179	–1·543	2·017
Model set 2: Adjusted for all covariates and local food price index				
ONI (La Nina)	–6·196†	2·423	–2·862†	–0·622
SOI (La Nina)	–6·520*	–3·955*	0·697	–3·107
MEI (La Nina)	–1·546	–1·324	–2·147	0·766
Model set 3: Adjusted for all covariates and river level				
ONI: La Nina)	–6·12	0·819	–3·167*	–0·0854
SOI (La Nina)	–6·348*	–4·694*	0·787	–2·487
MEI (La Nina)	–3·29	–3·204	–1·264	2·106
Model set 4: Interaction term of La Nina*Gender, adjusted for all covariates and river levels.				
ONI × Female	4·729	–2·141	0·539	–10·11†
SOI × Female	0·788	–2·55	–0·351	–12·25*
MEI × Female	2·772	–3·725	–2·071	–11·03*

ENSO, El Niño Southern Oscillation; ONI, Oceanic Niiño Index; SOI, Southern Oscillation Index; MEI, Multivariate ENSO Index.

†*P* < 0·10; *P < 0·05; **P < 0·01; ***P < 0·001.

When ENSO exposure was examined with respect to intake in grams, small but significantly lower intakes of yucca and rice (7 and 3 grams, model set 1) were identified, but the results were not consistent across different ENSO indices. Generally, within an index (with/out food prices and river level), there is agreement of ENSO effect but across ENSO indices, there is no consistent agreement on amounts of food consumed (see online supplementary material, Supplemental Table 3). However, consistent with the results presented above on frequency of intake, an interaction of ENSO phase with child sex was identified with girls consuming 10–12 g less sugar (*P* < 0·05) than boys.

### Dietary diversity

**Fig. 3:**
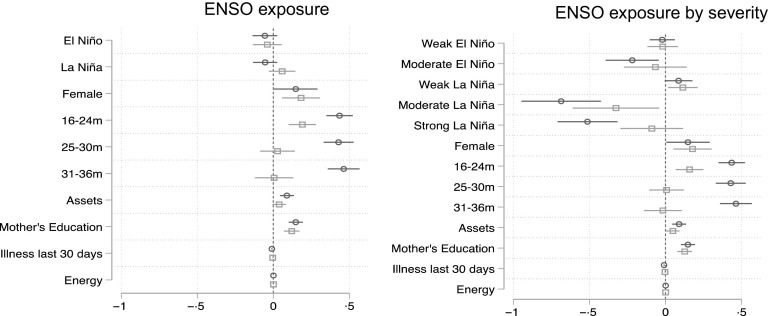
Random effects model results of ENSO exposure on dietary diversity score. ENSO, El Niño Southern Oscillation; MEI, Multivariate ENSO Index. 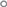, Bivariate; 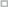, multivariate

Shown in Figure 3 is the summary of results from bivariate and multivariate model of ENSO exposure (MEI index and MEI severity index) on child DD score. Overall, general ENSO phases did not affect DD; however, severity of ENSO phases show varied effects on DD score. Girls had significantly higher DD score than boys by 0·18 (*P* < 0·01), but no significant interaction with ENSO events was detected (0·13 DD, *P* = 0·08). When evaluating ENSO using MEI severity, there was an increase in DD score (by 0·11, *P* < 0·05) during weak La Niña but reduced DD score observed during moderate La Niña by 0·32 (*P* < 0·05) food groups. DD was consistent across age groups and seasons with the median consumption of four food groups (interquartile range: 3, 5). There were no significant differences in DD score observed by birth order or income. Maternal education was strongly and positively associated with the DD score, whereas illness had a small but significant reduction in DD score.

On 22·4 % of days, children ate foods that were reported by the caregiver as gifts (24·1 % in girls compared to 21 % in boys). Similar to DD, the severity of ENSO exposures rather than generalised ENSO periods affected the consumption of donated food. Shown in Supplement Figure 3 are the results from the bivariate and multivariate Poisson models of MEI severity exposure and other factors associated with donated foods. Households with girls generally had 16 % higher consumption of donated foods compared to boys. During a moderate El Niño, gifting increased by 56 % (*P* < 0·05), as compared with a substantial reduction in the consumption of donated foods during moderate (67 %, *P* < 0·05) and strong La Niña. Consumption of donated foods was significantly associated with lower socio-economic status.

## Discussion

This study aimed to explore the associations between ENSO exposure and food consumption patterns in the Peruvian Amazon from 2010 to 2014. During 2011–12, severe floods were observed during La Niña, and as a result, an estimated half of the study population was temporarily displaced^(18)^. Under La Niña conditions, there was decreased consumption of grains, rice, dairy and sugar, and increased consumption of plantains. Interestingly, the reduced intake of staples such as grains/rice was accompanied by increases in the frequency of plantain consumption of 19–24 %, suggesting a potential substitution. In related research on food insecurity in this community, community fieldworkers described the substitution of yucca and plantains for rice during food insecure times^(23)^. There was no consistent (by any two ENSO indices) ENSO effect on the frequency of meals or snacks with animal source protein or fish. We posit that families may buffer their younger children from food insecurity; hence, no differences in intakes were observed except for sugar intake. This has been observed in other parts of Peru and in the Brazilian Amazon, where mothers provide nutritional buffering for their children, in particular for energy and protein^(35–37)^. Second, donating foods in this community provides an adaptive response to food security and chronic seasonal food scarcity, and this food sharing have been reported in other Amazonian communities as well^(13,38)^. Community fieldworkers have noted that in this community, it is acceptable to send older girls to other households to exchange household chores/services for food. This may explain the more frequent consumption of donated foods among girls, perhaps offsetting any gender differences previously observed in consumption of plantains, sugar and rice. The per cent of young girl children ever consuming donated foods was only 3 % higher compared to boys (21 %); thus, boy children are not excluded from consuming donated foods. A separate analysis (results not shown) found that consumption of donated foods increased the DD score significantly by 0·15 food groups (*P* < 0·05).

When the amounts of foods were examined, fewer grams of sugar and rice were consumed under La Niña, consistent with models that examined meals/snacks with sugar. For yucca, reduced intake was observed but the results were not consistent across ENSO indices. Although we hypothesised that fish intakes and meals with fish would be reduced under ENSO conditions, our results failed to bear this out. This may be due to several reasons: First, fish availability is known to be reduced during floods, but others have noted that fish availability increased after the 2011–2012 flooding because of access to new lakes with trapped fish that were created when the flood water receded^(38)^. Because of high market availability of fish, prices were lower, so households may have chosen to eat, rather than to sell, any fresh fish they obtained^(38)^. Second, families may have substituted canned tuna for fresh fish under food insecure conditions^(23)^. The estimated amount of fish consumed included amounts from canned products such as canned tuna and sardines, which are ubiquitous and less expensive alternative to fresh fish. Fishing is a source of livelihood in this community and fresh fish are often exported, domestically and internationally, representing a valuable source of income depending upon the size and variety of the fish^(17)^. In contrast, canned seafood is affordable, shelf-stable and convenient and possibly seasonally substituted for other sources of animal protein, causing overall consumption to remain relatively smooth throughout the year. Lastly, we modelled the amount consumed over zero grams so it possible there were more non-consumption during ENSO conditions that were not modelled.

There were several seasonal trends observed in food consumptions patterns. Seasonal variation in the intake of fish was confirmed in the panel regression models. Although there were no apparent seasonal trends in the frequency of consumption of food items with sugar, there were trends observed in the amounts of sugar consumed: significantly higher intakes were observed in June, July, September, October and December. The majority of the food items that contribute sugar to the child’s diet are ‘*refrescos’*, home-made fruit juices that contain on average of 21 g (about two tablespoons) of sugar per 100 ml cup of juice. In the dry season (June–November), there is a greater availability of fruits (particularly pineapple, star fruit, passion fruit, papaya, grapefruit, *camu camu* and watermelon), leading to greater sugar consumption during this period. Also, during the dry season, when the river levels are lower, there is increased trade and economic activity, potentially increasing household income and hence, access to fruits and sugar.

Despite seasonal fluctuations in the availability of produce and fish in this community, the DD score remained constant across seasons as children aged with minimal differences observed under various ENSO exposures. This is due to the seasonal patterns of food substitution and limitations in the scoring method due to the use of broad categories. For example, although fish consumption is seasonal, it is complemented by intakes of other meat such as chicken or canned tuna. Thus, shifts in the type of meat consumed within a year are not reflected in the DD score.

The study is limited by potential confounding due to secular trends in the regional food systems (distribution, introduction of new technology and trade); however, we are not aware of any food policy or programme that was implemented during the study period that may have affected the food intake in this community. Moderate El Niño and moderate La Niña occurred over very brief periods of 2 months total and therefore, associations that we have observed in the models for moderate ENSO events may not be robust and should be interpreted with caution. There are also several strengths to this study. First, we utilised longitudinal data from 252 children including dietary intake and morbidity records, over a period of 4 years. Second, we conducted sensitivity analysis with multiple ENSO indices to evaluate the effects of ENSO exposure on dietary patterns, DD score, amounts consumed and consumption of donated food items.

Many regions of the world are affected by one phase of the ENSO, but Peru is affected by both phases and severity. In Ecuador, El Niño-associated flood exposure *in utero* had a lasting negative impact on child cognitive outcomes years after exposure^(32)^. This same study illustrated ENSO floods affected outcomes through changes in income and child food consumption^(32)^. For example, children consumed significantly less servings of meat, fruits and vegetables, and grains^(32)^. Previous studies in Peru have shown that El Niño is associated with diarrhoeal incidence in Lima and reduced linear growth among children in coastal regions, and our findings suggest that La Niña affects dietary intake, which may negatively interact with differential burdens of diarrhoeal pathogens, resulting in greater-than-additive impacts on child linear growth^(18,39,^ ^40)^. Taken together, these studies and the presented analysis indicate that in addition to intra-annual (seasonal) shifts in diets and nutritional shifts due to weather, there are also inter-annual changes in diets and nutritional status due to weather also, which may be predicted and targeted through the use of ENSO indices.

This report evaluated the extent to which global measures of climate variability, mediated through local environmental conditions (river flow) and economy (local food prices), affect dietary intakes of children, thus quantifying these effects on the most vulnerable population. Our study adds to several notable qualitative studies in this region that have highlighted the negative effects between non-climate-related factors, such as changes in population, food system (availability of processed foods, market access and reliance on purchased foods), migration of male family members, social connections, economic activity (oil, logging, mining and cash-cropping produce for export), vector-borne diseases and climate-related factors such as variable seasonality and hydrology on food security in the region^(13,14,38,41)^. These changes coupled with low institutional capacity have resulted in communities with distinct profiles of vulnerability and similarly individualised capacities for resilience. Thus, climate-related policies in the Peruvian Amazon require localised tailoring with community input that not only targets immediate food availability during disaster conditions but also sustained food security through availability of flood-/drought-tolerant seeds, agriculture equipment and technology, and transparent systems of institutional and environmental monitoring^(13,14,38)^.

## Supplementary material

For supplementary material accompanying this paper visit https://doi.org/10.1017/S1368980020003705. click here to view supplementary material
