# Supplementary material for: La Niña weather impacts dietary patterns and dietary diversity among children in the Peruvian Amazon
Source: Public Health Nutr. 2020 Oct 27;24(11):3477–87. doi: 10.1017/S1368980020003705 (PMC8314922; doi:10.1017/S1368980020003705)
Supplement: Supplementary file 1 [file S1368980020003705sup001.docx]

**Supplemental Material**

**Table 1:** Monthly Food prices in Peruvian Soles (Loreto Region) from August 2010 to September 2014.

| Monthly Food prices in Peruvian Soles (Loreto Region) | Median (IQR) |
| --- | --- |
| Rice | 2.0 (1.9, 2.1) |
| Yucca | 1.2 (1.1, 1.6) |
| Plantain (*selva*) | 1.3 (1.1, 1.6) |
| White sugar | 2.1 (1.8, 2.3) |
| Eggs | 3.6 (3.2, 3.8) |
| Oil | 5.5 (5.0, 5.9) |
| Food price index of six food item above | 0.31 (-0.2, 1.5) |

Supplement Figure 1: Relationship between dietary patterns of children 9-36 months overlaid with monthly ENSO anomalies from three indices (MEI, ONI, SOI) from December 2010 to September 2014.


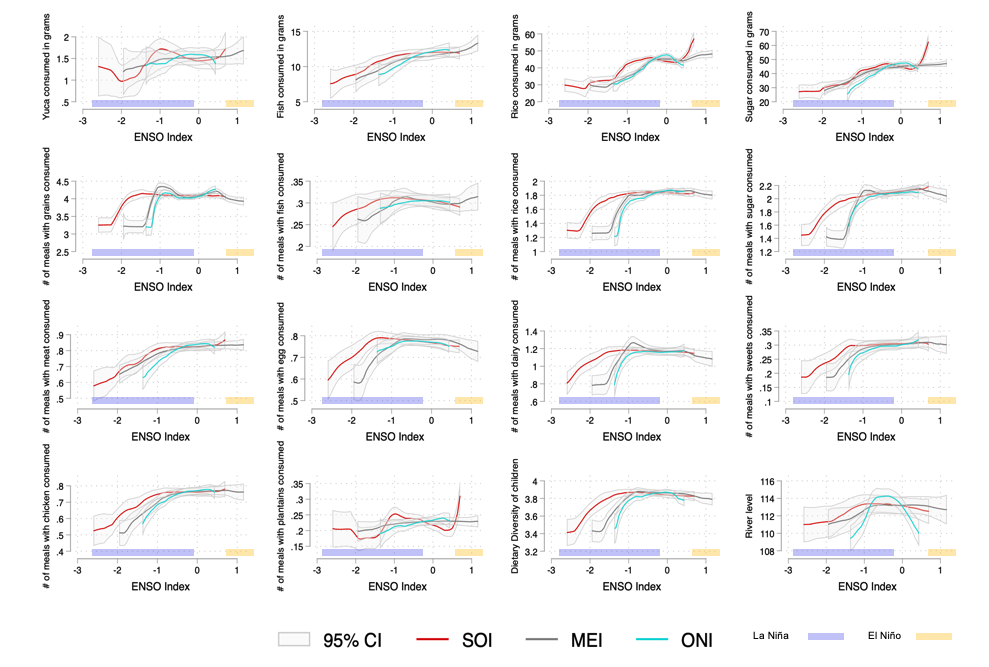


## Supplement Figure 2: Relationship between consumption patterns and river level

##
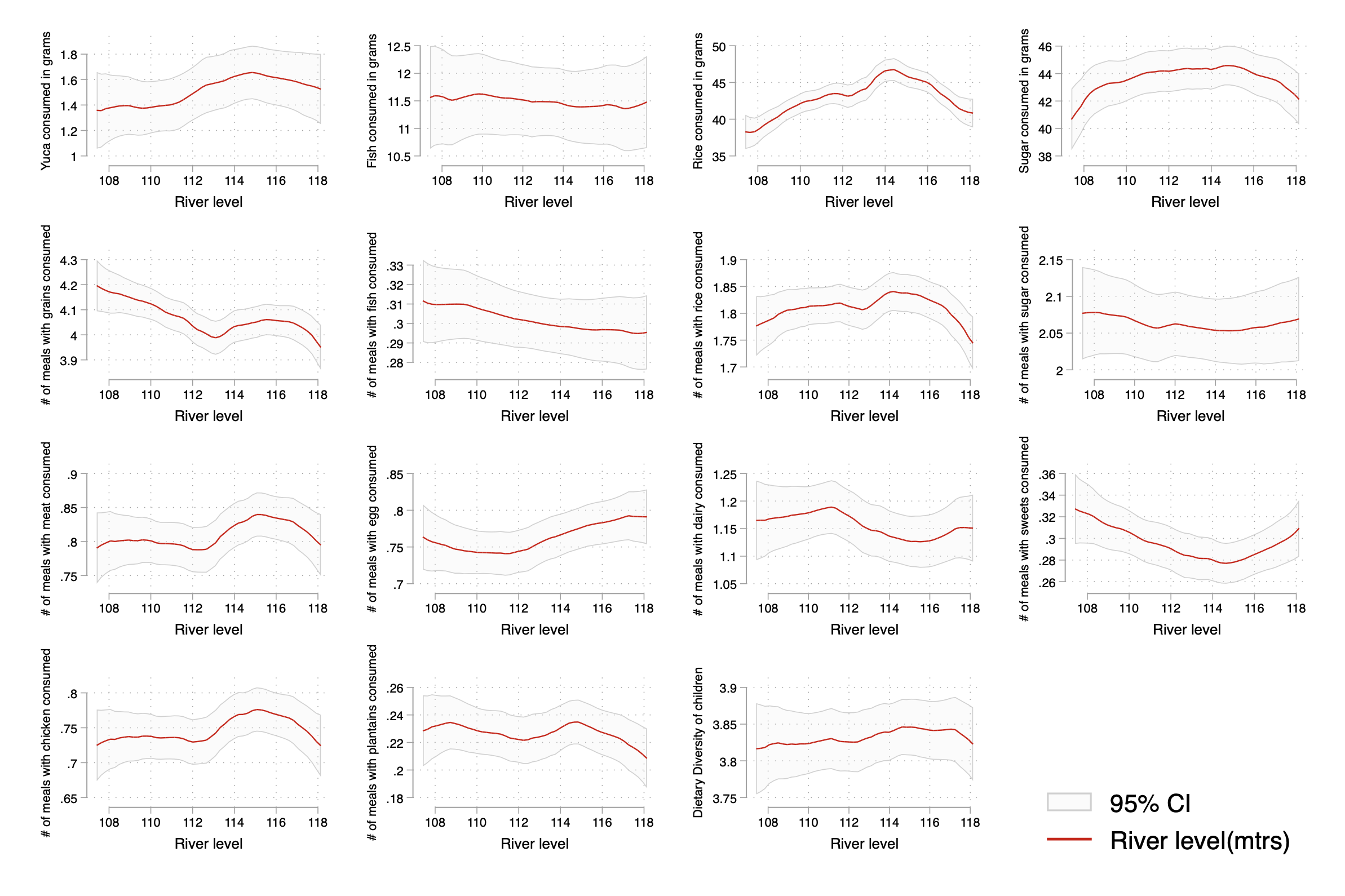


| Model set A | Fish | Grains | Meat | Eggs | Yucca | Chicken | Plantains | Rice | Dairy | Sugar |
| --- | --- | --- | --- | --- | --- | --- | --- | --- | --- | --- |
| ONI (La Nina) | 1.036 | 0.958* | 1.029 | 0.986 | 0.882 | 1.043 | 1.246** | 0.938* | 0.932* | 0.941* |
| SOI (La Nina) | 1.053 | 1.011 | 1.032 | 1.043 | 0.972 | 1.017 | 1.201*** | 0.988 | 0.979 | 0.986 |
| MEI (La Nina) | 0.969 | 0.963* | 1.055 | 0.972 | 1.045 | 1.056 | 1.195* | 0.942* | 0.981 | 0.971 |
|  |  |  |  |  |  |  |  |  |  |  |
| Model set B with local food price index |  |  |  |  |  |  |  |  |  |  |
| ONI (La Nina) | 1.038 | 0.961^*^ | 1.031 | 0.992 | 0.894 | 1.045 | 1.242^*^ | 0.946^+^ | 0.931^*^ | 0.942^*^ |
| Local food price index | 1.009 | 1.016^*^ | 1.008 | 1.033^+^ | 1.053 | 1.011 | 0.984 | 1.024^*^ | 1.014 | 1.019 |
| SOI (La Nina) | 1.055 | 1.015 | 1.034 | 1.05 | 0.981 | 1.019 | 1.198^*^ | 0.995 | 0.98 | 0.989 |
| Local food price index | 1.01 | 1.018^*^ | 1.009 | 1.036^*^ | 1.056 | 1.011 | 0.985 | 1.027^*^ | 1.013 | 1.019 |
| MEI (La Nina) | 0.97 | 0.969 | 1.06 | 0.982 | 1.076 | 1.062 | 1.187^*^ | 0.954 | 0.982 | 0.975 |
| Local food price index | 1.004 | 1.014^+^ | 1.013 | 1.029 | 1.06 | 1.015 | 0.985 | 1.023^*^ | 1.01 | 1.018 |
|  |  |  |  |  |  |  |  |  |  |  |
| Model set C with river level |  |  |  |  |  |  |  |  |  |  |
| ONI (La Nina) | 1.035 | 0.957* | 1.029 | 0.983 | 0.886 | 1.043 | 1.227** | 0.938* | 0.934^+^ | 0.942* |
| River level | 0.998 | 1 | 1.001 | 0.991 | 1.011 | 0.999 | 0.96 | 1.002 | 1.011 | 1.005 |
| SOI (La Nina) | 1.052 | 1.012 | 1.032 | 1.042 | 0.976 | 1.017 | 1.195*** | 0.989 | 0.979 | 0.987 |
| River level | 0.996 | 1.004 | 0.999 | 0.996 | 1.022 | 0.997 | 0.954 | 1.007 | 1.012 | 1.007 |
| MEI (La Nina) | 0.969 | 0.962* | 1.056 | 0.973 | 1.042 | 1.058 | 1.213** | 0.940* | 0.975 | 0.968 |
| River level | 0.997 | 1.003 | 0.996 | 0.994 | 1.02 | 0.993 | 0.940^*^ | 1.008 | 1.016 | 1.01 |
|  |  |  |  |  |  |  |  |  |  |  |
| Model set 4 interaction with gender, adjusted for river level |  |  |  |  |  |  |  |  |  |  |
| ONI x Female | 1.049 | 1.002 | 0.983 | 1.046 | 0.941 | 0.978 | 0.766* | 1.031 | 1.017 | 0.903* |
| SOI x Female | 1.01 | 1.002 | 0.994 | 0.956 | 0.801 | 0.988 | 0.815* | 1.005 | 1.077 | 0.911* |
| MEI x Female | 1.048 | 0.984 | 0.912 | 1.052 | 1.447^+^ | 0.878^+^ | 0.837 | 0.998 | 1.039 | 0.903* |

Supplement table 2: Poisson panel regression results examining ENSO exposure on food items consumed by children 9-36 months. All models adjusted for gender, age, parity, seasons (months), assets, energy(kcal), household income, maternal education, and illness in the previous 30 days. Models 2 adjusted for local food prices (index) of six foods. Models 3 adjusted additional for median river level (meters). Model 4 presents the interaction term and also adjusted for river levels meters).

Exponentiated coefficients; ^+^ *p* < 0.10, * *p* < 0.05; ** *p* < 0.01; *** *p* < 0.001;

Supplement table 3: Panel regression of ENSO exposure in intake of fish, yucca, plants, rice in grams. All models adjusted for gender, age, parity, seasons (months), assets, energy(kcal), household income, maternal education, and illness in the previous 30 days. Models 2 adjusted additional for median river level (meters). Model 3 presents the interaction term and also adjusted for river levels (meters).

| Model set 1 adjusted for all covariates | Yuca | Fish | Rice | Sugar |
| --- | --- | --- | --- | --- |
| ONI (La Nina) | -7.384^*^ | 0.517 | -2.632^+^ | 0.0177 |
| SOI (La Nina) | -7.165^*^ | -4.719^*^ | 0.978 | -2.372 |
| MEI (La Nina) | -3.287 | -3.179 | -1.543 | 2.017 |
|  |  |  |  |  |
| Model set 2 adjusted for all covariates and local food price index |  |  |  |  |
| ONI (La Nina) | -6.196^+^ | 2.423 | -2.862^+^ | -0.622 |
| Local food price index | 1.1 | 1.788^*^ | -1.243^*^ | -2.742^*^ |
| SOI (La Nina) | -6.520^*^ | -3.955^*^ | 0.697 | -3.107 |
| Local food price index | 1.303 | 1.209^+^ | -1.152^*^ | -2.877^*^ |
| MEI (La Nina) | -1.546 | -1.324 | -2.147 | 0.766 |
| Local food price index | 1.508 | 1.541^*^ | -1.378^*^ | -2.674^*^ |
|  |  |  |  |  |
| Model set 3, adjusted for all covariates and river level |  |  |  |  |
| ONI: La Nina) | -6.12 | 0.819 | -3.167^*^ | -0.0854 |
| River level | 2.319 | 0.738 | -1.653^*^ | -0.329 |
| SOI (La Nina) | -6.348^*^ | -4.694^*^ | 0.787 | -2.487 |
| River level | 2.689^+^ | 0.14 | -1.462^*^ | -0.895 |
| MEI (La Nina) | -3.29 | -3.204 | -1.264 | 2.106 |
| River level | 2.754^+^ | 0.663 | -1.425^*^ | -0.405 |
|  |  |  |  |  |
| Model set 4; Interaction term of La Nina *Gender, adjusted for all covariates and river levels. |  |  |  |  |
| ONI x Female | 4.729 | -2.141 | 0.539 | -10.11^+^ |
| SOI x Female | 0.788 | -2.55 | -0.351 | -12.25^*^ |
| MEI x Female | 2.772 | -3.725 | -2.071 | -11.03^*^ |

^+^ *p* < 0.10, * *p* < 0.05; ** *p* < 0.01; *** *p* < 0.001;

Supplement Figure 3: Poisson regression models results on factors associated with consumption of gifted foods
